# Controlled Unusual Stiffness of Mechanical Metamaterials

**DOI:** 10.1038/srep20312

**Published:** 2016-02-03

**Authors:** Wooju Lee, Da-Young Kang, Jihwan Song, Jun Hyuk Moon, Dongchoul Kim

**Affiliations:** 1 Department of Mechanical Engineering, Sogang University, Seoul 04107, Korea; 2 Department of Chemical and Biomolecular Engineering, Sogang University, Seoul 04107, Korea

## Abstract

Mechanical metamaterials that are engineered with sub-unit structures present unusual mechanical properties depending on the loading direction. Although they show promise, their practical utility has so far been somewhat limited because, to the best of our knowledge, no study about the potential of mechanical metamaterials made from sophisticatedly tailored sub-unit structures has been made. Here, we present a mechanical metamaterial whose mechanical properties can be systematically designed without changing its chemical composition or weight. We study the mechanical properties of triply periodic bicontinuous structures whose detailed sub-unit structure can be precisely fabricated using various sub-micron fabrication methods. Simulation results show that the effective wave velocity of the structures along with different directions can be designed to introduce the anisotropy of stiffness by changing a volume fraction and aspect ratio. The ratio of Young’s modulus to shear modulus can be increased by up to at least 100, which is a 3500% increase over that of isotropic material (2.8, acrylonitrile butadiene styrene). Furthermore, Poisson’s ratio of the constituent material changes the ratio while Young’s modulus does not influence it. This study presents the promising potential of mechanical metamaterials for versatile industrial and biomedical applications.

Recent advances in the fabrication of micro-/nano-structures have resulted in the development of interesting mechanical metamaterials like phononic (acoustic)^1^ and meta-fluidic materials^2^. Phononic metamaterials have a negative effective bulk modulus^3^ or negative effective mass densities^4^. These properties can be used in applications like acoustic subwavelength imaging^5^, superlensing^6^, and transforming acoustics^7^,^8^. Meta-fluidic materials have extremely large bulk modulus compared to shear modulus, which makes them behave like a fluid^2^. Similar concepts have also been explored to improve mechanical characteristics with new designs of micro lattices without changing the chemical composition or weight^9^,^10^. Ultralight and ultrastiff metamaterials have been reported, which maintain a nearly constant stiffness per unit mass density even at ultralow densities^10^. Anisotropic versions of meta-fluidic materials have been suggested, which can be used in elasto-dynamic cloaking devices^11^. Wave trapping in cellular metamaterial plates constructed by bending-dominated and stretch-dominated unit-cells has been studied based on the numerical simulation^12^.

The interesting mechanical properties of mechanical metamaterials are a consequence of their deliberate structuring, and not of the bulk material that comprises them. Owing to the complicated patterns involved^10^,^13^, currently mechanical metamaterials are mostly fabricated by using three dimensional printing. Recently, triply periodic bicontinuous structures have received attention because their geometric characteristics can be precisely controlled in the sub-micro scale with various fabrication methods such as block-copolymer self-assembly^13^, phase-mask lithography^14^, and multi-beam interference lithography^15^,^16^,^17^. For example, when the structure is fabricated by block-copolymer self-assembly, the volume fraction of each component is a key parameter that determines the thickness and aspect ratio of the structure. When the structure is fabricated by lithography, the thickness and aspect ratio of the structure can be controlled by the exposure time and the incident angle of the laser, respectively. To this end, triply periodic bicontinuous structures have been studied for three dimensional photonic crystals because of their wide and complete photonic band gaps^18^,^19^. However, they are not considered as mechanical metamaterials even though their sub-unit structure can be finely tuned.

In this report, we study the potential of triply periodic bicontinuous structures as mechanical metamaterials. The sub-unit structure of the representative triply periodic bicontinuous structures, which include Schwarz’ primitive, diamond, and Schoen’s gyroid structures, are systematically investigated to present a highly versatile mechanical metamaterial with a notably wide range of the ratios of elastic moduli. The effect of the material properties of the constituent material on the mechanical properties is also studied.

## Results and Discussion

**Figure 1:**
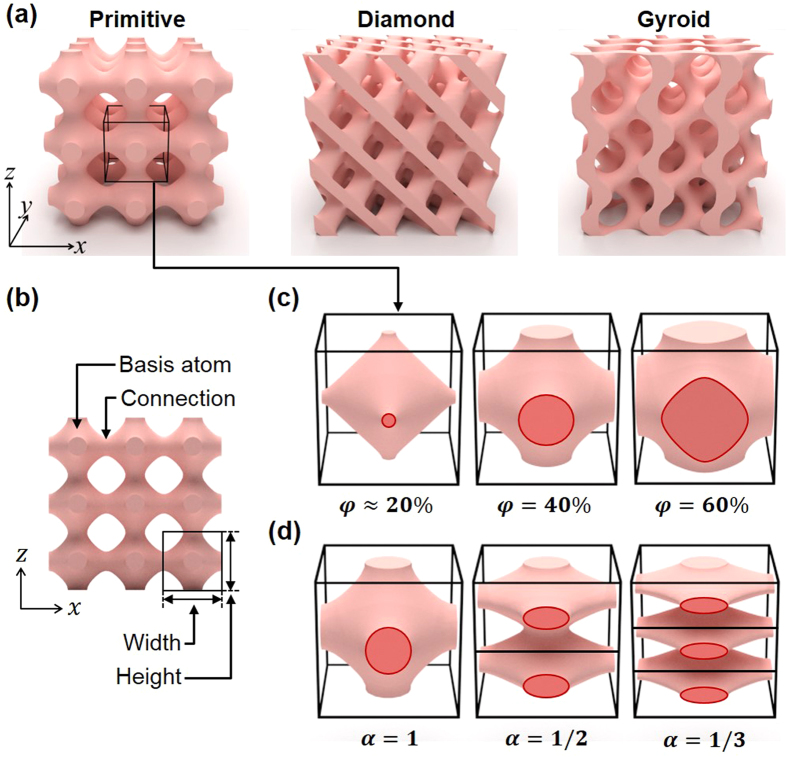
The representative triple periodic bicontinuous structures (**a**) Illustrations of representative triply periodic bicontinuous structures, including Schwarz’ primitive (*P*), diamond (*D*) and Schoen’s gyroid (*G*) structures. (**b**) Basis atom and connection parts of *P* structure. Width and height of unit cell is measured to calculate aspect ratio. Morphological changes of *P* structure with respect to the (**c**) volume fraction (*φ*) ranging from about 20% to 60%, and (**d**) aspect ratio (*α*) ranging from 1 to 1/3.

Triply periodic bicontinuous structures were defined by a volume enclosed by triply periodic minimal surfaces^20^,^21^,^22^. The representative triply periodic bicontinuous structures, including Schwarz’ primitive (*P*), diamond (*D*), and Schoen’s gyroid (*G*), are illustrated in Fig. 1a. Literature describes the *P*, *D*, and *G* structures by using the level surfaces of the following trigonometric functions^23^,^24^,^25^:


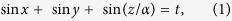



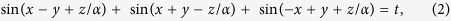



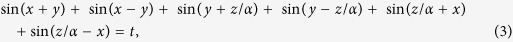


where *t* and *α* stand for the threshold of level surface and aspect ratio, respectively. The threshold determines a volume fraction (*φ*) that is defined by the ratio of the volume enclosed by the level surface to the volume occupied by the structure. The structures consist of basis atoms and connections between basis atoms (Fig. 1b). The aspect ratio is defined by the ratio of the lattice constant along the tensile to shear direction. We choose the volume fraction (*φ*) and aspect ratio (*α*) as geometric variables that characterize the geometry of structures. Figure 1c shows the various geometries of *P* structures with respect to different volume fractions (20%, 40%, and 60%). When the structure is fabricated by a photolithography process, the volume fraction of the structure defined by trigonometric functions is determined by the exposure time and intensity of the laser. During the crosslinking reaction of negative photoresist (SU8), the cross-linked part of the photoresist can be reduced as the exposure time and intensity of the laser decrease. As the exposure time and intensity of the laser decrease, the volume fraction of the structure also decreases and the structure can collapse due to the diminished connections between the basis atoms (pinch-off)^17^. The volume fractions of *P* and *D* structures are about 20% and that of *G* structure is about 5% at pinch-off 26. Figure 1d shows the geometry of *P* structures with respect to the different aspect ratios (1, 1/2, and 1/3). When the aspect ratio is unity, the structure has an identical shape along the *x*, *y*, and *z*-directions. By changing the aspect ratio, the structure becomes a transverse isotropic structure.

**Figure 2:**
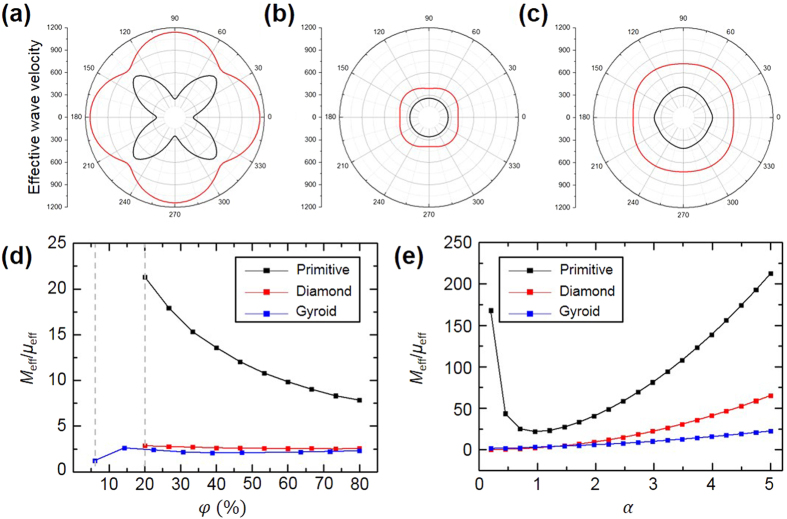
Effective mechanical properties of the triply periodic bicontinuous structures (**a–c**) Effective elastic wave velocities (transverse: black solid line, longitudinal: red solid line) of *P*, *D*, and *G* structures along different directions where the volume fraction and aspect ratio are 20% and unity, respectively. (**d**) The ratios of effective longitudinal modulus to shear modulus (*M*_eff_/*μ*_eff_) along 90^o^ directions as a function of the volume fraction. (**e**) The *M*_eff_/*μ*_eff_ ratios of the structures versus the aspect ratio.

In elastic mechanics, the stiffness of a structure can be characterized by elastic wave velocities. To investigate the stiffness of the triply periodic bicontinuous structures (*i.e*., *P*, *D*, and *G*) with the propagation of elastic waves, we have calculated the effective wave velocity of each structure under long wavelength condition. The effective longitudinal and transverse wave velocities of *P*, *D*, and *G* structures in different directions are shown in Fig. 2a–c, where the 0^o^ and 90^o^ represent *x* and *z*-directions, respectively. Simulations show that the effective wave velocity of *P* structure has much greater variation along with different directions than those of *D* and *G* structures. Specifically, in the 0^o^ and 90^o^ of directions of *P* structure, the longitudinal wave velocity is about 400% greater than the transverse wave velocity. It indicates that the *P* structure is the most anisotropic structure among *P*, *D*, and *G* structures. Then, we have retrieved the effective elastic parameters. The triply periodic bicontinuous structures are fabricated by the interference lithography, and only the direction of 90^o^ with respect to a substrate is practically allowable in applications. Thus, we calculated the ratio of longitudinal modulus (*M*_eff_) to shear modulus (*μ*_eff_) only in the direction of 90^o^ to compare the behaviors of effective elastic parameters according to the changes of geometric variables. As shown in Fig. 2d, the *M*_eff_/*μ*_eff_ ratio of *P* structure is significantly increased as the volume fraction of structure decreases, while those of *D* and *G* structures show slight changes. We also investigated the effect of aspect ratio on the *M*_eff_/*μ*_eff_ ratios of structures (Fig. 2e). It clearly reveals that the *P* structure have wider attainable range of *M*_eff_/*μ*_eff_ ratio than others. Interestingly, the *M*_eff_/*μ*_eff_ ratio of *P* structure is increased drastically as the aspect ratio decreases below the unity. It is caused by the distinctive trend of longitudinal wave velocity of *P* structure with respect to the change of aspect ratio (see Supplementary Information).

**Figure 3:**
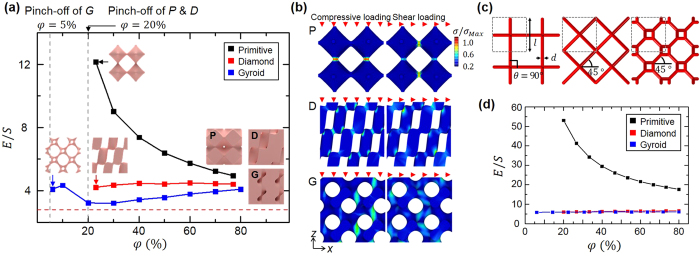
The numerical simulation of effect of volume fraction on elastic moduli (**a**) Effect of volume fraction (*φ*) on the *E*/*S* ratios and corresponding geometries of structures. Red dashed line describes the *E*/*S* ratio of homogeneous material (2.8). All simulations were carried out above the minimum volume fraction where the pinch-off occurs. (**b**) Stress distributions of *P*, *D*, and *G* structures under compressive loading along the *z*-direction and shear loading along the *x*-direction, respectively. For visualization, the stress is normalized by the maximum stress. (**c**) Simplified *P*, *D*, and *G* structures as beams with connection angle (*θ*) and diameter (*d*). (**d**) Analytical calculation of *E*/*S* ratio of simplified *P*, *D*, and *G* structures as a function of the volume fraction. The ranges of volume fraction are selected to be same with those of numerical models.

In order to study the stiffness of structures with their elastic moduli, the ratios of Young’s modulus to shear modulus in the direction of 90^o^ according to the changes of geometric variables are investigated. First, the ratios of Young’s modulus to shear modulus (*E*/*S* ratio) of *P*, *D*, and *G* structures as functions of volume fraction are calculated, and are shown in Fig. 3a. *E*/*S* ratios of *D* and *G* structures does not change from around 4.4 and 3.7 even though the volume fractions of *D* and *G* structures increase considerably from 20% to 80% and from 5% to 80%, respectively. Interestingly, *E*/*S* ratio of *P* structure increases significantly as the volume fraction decreases from 80% to 20%. When the volume fraction of *P* structure becomes about 20%, *E*/*S* ratio increases to 12, which is about 300% larger than that of isotropic bulk material, which is 2.8. In this case, the Young’s modulus and shear modulus of *P* structure decrease about 90%, as the volume fraction decreases from 80% to 20%. To understand the distinguishing *E*/*S* ratio of *P* structure, stress analysis is performed as shown in Fig. 3b. It shows that stress is concentrated only on connections between basis atoms regardless of the structure type and the loading direction. When the loading is applied on *D* and *G* structures, compressive and shear stress are always generated on the connections regardless of the loading direction. In case of *P* structures, however, a compressive stress is generated only at connections in directions parallel to the loading direction as the compressive loading is applied.

However, stress is generated in all the connections when shear loading is applied. Thus, we simplify the *P*, *D*, and *G* structures by beam structures shown as red beams in Fig. 3c^27^. Beams can be described by diameter (*d*) and connection angle (*θ*). The connection angle is defined as the angle between beams and the *xy*-plane where the loading is applied. The connection angle of the *P* structure is 90^o^ and those of *D* and *G* structures are 45^o^. We calculate deflections of beams under compressive and shear loadings by using Euler-Bernoulli beam theory^28^ According to the beam theory, the deflections of beams under compressive and shear loadings are inversely proportional to the second and the forth power of beam diameter (*d*), respectively. Thus, the deflection under shear loading is more significantly affected by the change of beam diameter than that under compressive loading. When the connection angle is 45^o^, like it is for *D* and *G* structures, deflections of beams under compressive and shear loadings becomes consistent regardless of the diameter of beams (see Supplementary Information Eq. S1). When the connection angle is 90^o^, however, the ratio of deflections from compression and shear loading in the *P* structure increases and presents a high *E*/*S* ratio as the diameter of beams decreases because the deflection under shear loading is more dependent on the change of beam diameter (see Supplementary Information Eq. S2). Figure 3d presents the calculated *E*/*S* ratios with respect to the volume fraction. It shows that the *E*/*S* ratio of simplified *P* structure increases up to 53 as the volume fraction decreases to 20% while those of *D* (6.4) and *G* (6.0) structures are not changed relatively.

**Figure 4:**
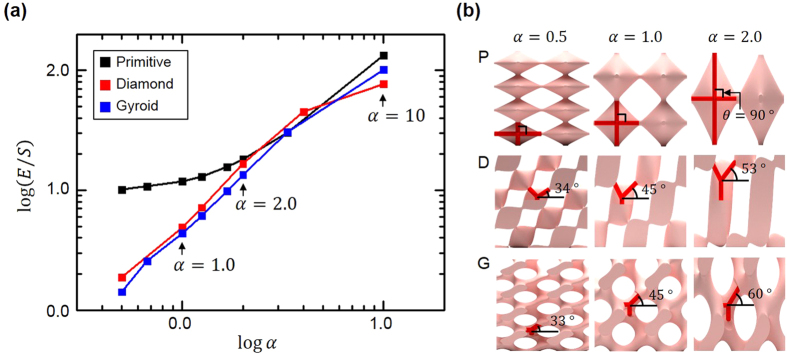
The numerical simulation of effect of aspect ratio on elastic moduli (**a**) Effect of aspect ratio on mechanical anisotropy with various structures. All variables are plotted in log scale. (**b**) Simplified *P*, *D*, and *G* structures with different connection angles of beams correspond to the various aspect ratio (0.5, 1.0, and 2.0).

The effect of the aspect ratio, which is the other variable, on the *E*/*S* ratio is investigated as shown in Fig. 4a. As the aspect ratio goes far from unity, the triply periodic bicontinuous structures becomes transverse isotropic along the *z*-direction. Thus, we measured the Young’s modulus and shear modulus only along the *z*-direction to focus on the variation of *E*/*S* ratio. As the aspect ratio increases, the *E*/*S* ratio of *P*, *D*, and *G* structures increase monotonically. By increasing the aspect ratio from 0.5 to 10, the *E*/*S* ratio of *P*, *D*, and *G* structures can be increased about 1200%, 5000%, and 7000%, respectively. Interestingly, only the *E*/*S* ratio of *P* structure decreases slightly compared to those of *D* and *G* structures as the aspect ratio decreases to values smaller than 2.0. When the aspect ratio decreases from 2.0 to 0.5, the *E*/*S* ratio of the *P* structure decreases from 18 to 10 while that of *D* and *G* structures significantly decreases from 17 to 1.5 and from 13 to 1.4, respectively. When the aspect ratio decreases, the connection angles (*θ*) of *D* and *G* structures are decreased, which become almost parallel to the *xy*-plane, while that of *P* structure is not changed because the connections of *P* structure are vertical to the *xy*-plane (Fig. 4b). Thus, *D* and *G* structures come to be relatively weaker under compressive loading compared to under shear loading due to the absence of connections vertical to the *xy*-plane. The connection of *P* structure becomes relatively strong under compressive loading compared to that under shear loading. This induces larger *E*/*S* of the *P* structure compared to those of *D* and *G* structures.

**Figure 5:**
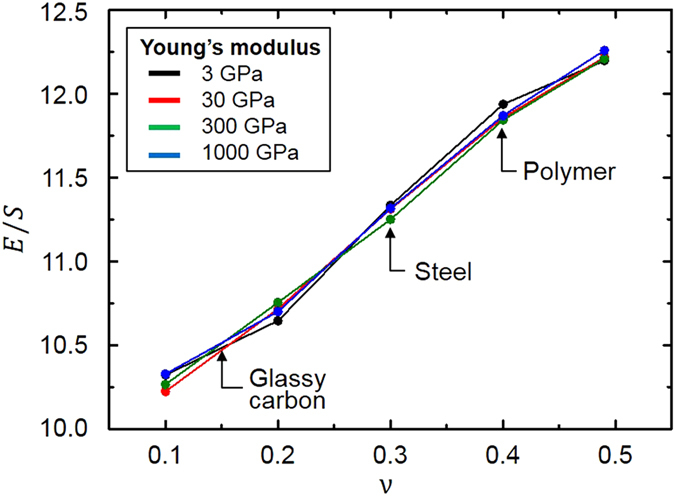
The *E*/*S* ratio of *P* structure as a function of Poisson’s ratio with respect to the different Young’s moduli of constituent material.

The effect of the material property of the base material on the *E*/*S* ratios is investigated as shown in Fig. 5. We consider Young’s modulus and Poisson’s ratio of the base material ranging from 3 GPa to 1000 GPa and from 0.1 to 0.49, respectively. We consider the *P* structure whose ratio of effective mechanical properties changes considerably according to the volume fraction. The volume fraction and aspect ratio are set to 20% and unity, respectively. The result shows that the change of *E*/*S* ratios is negligible when Young’s modulus of constituent material increases from 3 GPa to 1000 GPa. However, the ratio of the *P* structure increases 18.8% as Poisson’s ratio increases from 0.1 to 0.49. The change of Young’s modulus of the base material influences the deflections of the *P* structure under compressive and shear loadings equally. For example, when Young’s modulus increases 100%, both the deflections of the *P* structure under compressive and shear loadings decrease by 50%. Thus, the *E*/*S* ratios is not affected by Young’s modulus of the base material. In general, with a base material that has a large Poisson’s ratio, a large lateral deformation is generated under compressive loading. In triply periodic bicontinuous structures, however, the lateral deformation of unit cell is interfered by that of neighboring unit cells due to the periodicity of the structure, which induces more resistance to the axial deformation under compressive loading. Thus, simulation results also show that Young’s modulus of *P* structure increases as Poisson’s ratio of the base material increases. On the other hand, the deformation under shear loading is independent of Poisson’s ratio. As a result, the *E*/*S* ratio of *P* structure increases as Poisson’s ratio of the base material increases.

## Conclusion

In conclusion, we have demonstrated the remarkable range of stiffness of triply periodic bicontinuous structures for mechanical metamaterials. The stiffness of triply periodic bicontinuous structures is studied with elastic wave velocity and elastic moduli. The effects of geometric variables (volume fraction and aspect ratio) and material properties (Young’s modulus and Poisson’s ratio) on the stiffness of triply periodic bicontinuous structures are investigated. The simulation results show that *M*_eff_/*μ*_eff_ ratio and *E*/*S* ratio of *P* structure is most dependent on the change of the geometric variables. The *M*_eff_/*μ*_eff_ ratio and *E*/*S* ratio of *P* structure are increased by about 170% and 150%, respectively, when the volume fraction decreases from about 80% to 20%. The aspect ratio can significantly change the *E*/*S* ratios of *P*, *D*, and *G* structures by about 1200%, 5000%, and 7000% as the aspect ratio increases from 0.5 to 10, respectively. Poisson’s ratio of the base material is also found to be critical for the *E*/*S* ratios, which increases about 20% as the Poisson’s ratio increases from 0.1 to 0.49. We believe that the fabrication of triply periodic bicontinuous structures with guided geometric variables and materials makes it possible to design functional materials with unusual mechanical properties.

## Methods

### Calculation of longitudinal and shear modulus in long wavelength condition

The effective wave velocity with respect to the volume fraction and aspect ratio was calculated to investigate the effect of geometric variables on the propagation of elastic waves. The effective longitudinal and transverse wave velocities in all direction were calculated by using ***v***** = *****w***/***k*** where ***k**→*0 (long wavelength condition), where ***w*** and ***k*** are the angular frequency and the wave vector. The effective longitudinal modulus 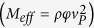 and the effective shear modulus 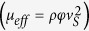 were obtained from the effective wave velocity of longitudinal (*v*_P_) and transverse wave (*v*_*S*_) with its material density (*ρ*) and the volume fraction (*φ*), respectively. In these simulations, material density, longitudinal wave velocity, and transverse wave velocity were set to be 1050 kg/m^3^, 2474 m/s, and 1010 m/s, respectively. For simplicity, we considered simplified structures with a single unit cell.

### Calculation of Young’s modulus and shear modulus

In order to investigate the effect of geometric variables on the mechanical properties of representative triply periodic bicontinuous structures (*P*, *D*, and *G*), we calculated the ratio of Young’s modulus to shear modulus (*E*/*S*) with various values of the volume fraction and aspect ratio. The minimum volume fractions of *P*, *D*, and *G* structures were set to be about 20%, 20%, and 5%, respectively, to avoid their ‘pinch-off’ that cause the collapse of structures. The maximum volume fractions were selected to be about 80% from the suggested possible maximum volume fractions of triply periodic bicontinuous structures^26^. The aspect ratio was set to range from 0.5 to 10 for all structures, which is wide enough to cover the practically possible ranges of aspect ratio of triply periodic bicontinuous structures fabricated with interference lithography. Material properties of a typical polymer (*e.g*. acrylonitrile butadiene styrene (ABS)) were employed. It has a Young’s modulus of 3.0 GPa and a Poisson’s ratio of 0.4. The Young’s modulus and shear modulus were calculated by simulating deflections under compressive and shear loadings along the *z*-direction. Fixed boundary condition was applied on the bottom of structures. The lateral surfaces of structures were set to be free. The magnitude of loading was controlled to ensure that the elastic strain of structures is less than 1%. A finite element analysis was carried out for accurate and efficient calculations by using the implicit solver of LS-DYNA (LSTC). Based on level surfaces in equations (1, 2, 3), the volumetric meshes of structures were generated. Each finite element model consists of at least 2 × 2 × 2 unit cells and 1.5 million elements.

## Additional Information

**How to cite this article**: Lee, W. *et al*. Controlled Unusual Stiffness of Mechanical Metamaterials. *Sci. Rep*. **6**, 20312; doi: 10.1038/srep20312 (2016).

## Supplementary Material

Supplementary Information
