# Supplementary material for: Controlled Unusual Stiffness of Mechanical Metamaterials
Source: Sci Rep. 2016 Feb 3;6:20312. doi: 10.1038/srep20312 (PMC4738250; doi:10.1038/srep20312)
Supplement: Supplementary Information [file srep20312-s1.pdf]

Supplementary Information

Controlled Unusual Stiffness of Mechanical Metamaterials

Wooju Lee[1], Da-Young Kang[2], Jihwan Song[1], and Jun Hyuk Moon[2,*], Dongchoul Kim[1,*]

[1]Department of Mechanical Engineering, Sogang University, Seoul 121-742, Korea.
[2]Department of Chemical and Biomolecular Engineering, Sogang University, Seoul 121-742, Korea.
*Corresponding Author: (Jun Hyuk Moon) Electronic mail: junhyuk@sogang.ac.kr; (Dongchoul Kim) Electronic mail: dckim@sogang.ac.kr;

Table of Contents:

1. **Analytical implementation of the deflection of beam**
   - Deflection of beam in *D* and *G* structures
   - Deflection of beam in *P* structure
2. **Numerical simulation details**
   - Effective wave velocities
   - Effect of aspect ratio on dynamic moduli
   - Boundary conditions in finite element analysis

1. Analytical implementation of the deflection of beam

In order to analytically assess the deflection of beam in triply periodic bicontinuous structures under compressive and shear loadings, we employ Euler-Bernoulli beam theory.[1] In the Euler-Bernoulli beam theory, the deflections of beam under compressive and shear loadings are defined to be inversely proportional to the square and the forth power of beam diameter, respectively. When the direction of loading is parallel to the axial direction of beam, the compressive (δ_{c}) and shear (δ_{s}) deflections are expressed as $\delta_{\mathrm{c}} = 4Fl/\pi E_0 d^2$ and $\delta_{\mathrm{s}} = 64Fl^3/3\pi E_0 d^4$, respectively. F is magnitude of applied loading, l, d are length and diameter of beam, and E_0 is Young's modulus of base material, respectively. The cross sectional shape of beam is assumed to be a circle. The theory shows that the deflection under shear loading is more affected by the change of beam diameter compared to that under compressive loading.

Deflection of beam in *D* and *G* structures

When loading is applied on D and G structures, the loading are divided into two components, the direction of axial force (F_{A}) and lateral force (F_{L}) which are parallel and perpendicular to the axial direction of beam, respectively. The magnitude of F_{A} and F_{L} are same regardless of the direction of

loading (*i.e.*, F_A=F_L=$F/\sqrt{2}$), because the connection angle of beam in *D* and *G* structures is $45\,°$. When the compressive loading is applied on *D* and *G* structures, the axial deflections (δ_A) and lateral deflections (δ_L) of can be expressed as $\delta_A = 4F_A l/\pi E_0 d^2$ and $\delta_L = 64F_L l^3/3\pi E_0 d^4$, respectively. Then, the deflection of *D* and *G* structures can be calculated by summation of δ_A and δ_L along the direction parallel to the compressive loading, that is $\delta_c = 2Fl/\pi E_0 d^2 + 32Fl^3/3\pi E_0 d^4$. In the same manner, deflection of *D* and *G* structures under shear loading can be calculated as $\delta_s = 2Fl/\pi E_0 d^2 + 32Fl^3/3\pi E_0 d^4$. Therefore, the ratio of deflections from compressive and shear loading (δ_c/δ_s) becomes consistent regardless of the diameter of beams as follows:

$$\frac{\delta_c}{\delta_s} = \left(\frac{2Fl}{\pi E_0 d^2} + \frac{32Fl^3}{3\pi E_0 d^4}\right) / \left(\frac{2Fl}{\pi E_0 d^2} + \frac{32Fl^3}{3\pi E_0 d^4}\right) = 1 \qquad \text{(S1)}$$

The ratio of Young's modulus to shear modulus (*E*/*S*) of structure is linearly proportion to the ratio of deflections from compressive and shear loading (δ_c/δ_s). As a result, the *E*/*S* of *D* and *G* structures can be consistent regardless of the volume fraction.

Deflection of beam in *P* structure

The connection angle of simplified beams in *P* structure is $90°$. Thus, the magnitudes of F_A and F_L in *P* structure are changed with respect to the direction of loading. When the compressive loading is applied on *P* structure, axial force (F_A) is equal to F and lateral force (F_L) is zero, while $F_A = 0$ and $F_L = F$ as the shear loading is applied. According to the Euler-Bernoulli beam theory, the axial (δ_A) and lateral (δ_L) deflections of beam in *P* structures under compressive and shear loading can be expressed as $\delta_A = 4Fl/\pi E_0 d^2$ and $\delta_L = 64Fl^3/3\pi E_0 d^4$, respectively. Because the directions of deflection and loading are always same, the deflection of *P* structures can be calculated by $\delta_c = \delta_A$ and $\delta_s = \delta_L$. Therefore, the ratio of deflections from compressive and shear loading is proportion to the square of diameter.

$$\frac{\delta_c}{\delta_s} = \left(\frac{4Fl}{\pi E_0 d^2}\right) / \left(\frac{64Fl^3}{3\pi E_0 d^4}\right) \propto d^2 \qquad \text{(S2)}$$

As a result, the *E*/*S* of *P* structures is increased as the volume fraction decreases.

2. Numerical simulation details

Effective wave velocities

The effective longitudinal and transverse wave velocities were calculated as a function of geometric variables with respect to the various structures. All calculations was based on the long wavelength condition where the wavelength (λ=100L) is much larger than the size of unit cells. The effective wave velocity was calculated by using $\boldsymbol{v}=\boldsymbol{w}/\boldsymbol{k}$ where $\boldsymbol{k}\rightarrow 0$. As shown in figure S1, when the volume fraction changes, the effective transverse wave velocities of *P*, *D*, and *G* structures have similar range while the longitudinal wave velocity of *P* structure is much greater than those of other structures. When the aspect ratio is changed, *P* structure shows a relatively little variation of the effective longitudinal wave velocity compared to those of *D* and *G* structure.

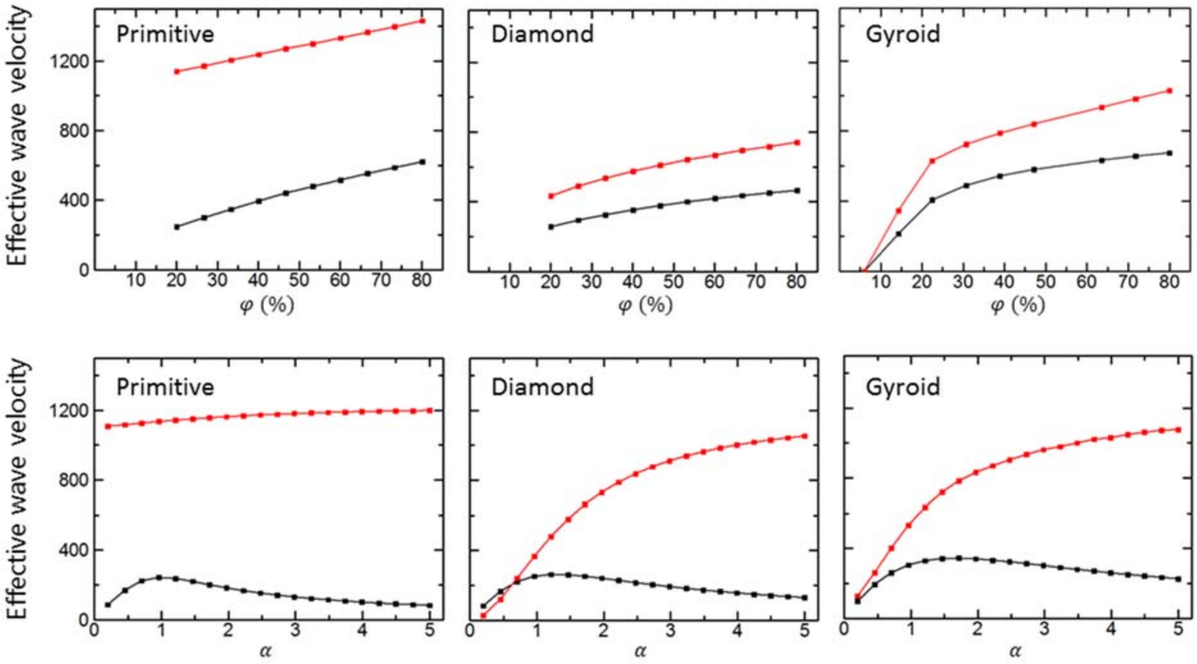

Figure S1. Effective wave velocities (transverse wave: black line, longitudinal wave: red line) of *P*, *D*, and *G* structures as a function of volume fraction and aspect ratio.

Effect of aspect ratio on dynamic moduli

For more sophisticated analysis of the effect of aspect ratio, we have calculated the elastic wave velocity of the structures along various directions (0 °, 45 °, and 90°). Figure S2 shows the ratio of longitudinal modulus to shear modulus (M_{eff}/μ_{eff} ratio) under long wavelength condition along different direction of structures. Interestingly, simulation results show that the M_{eff}/μ_{eff} ratio of *P* structure along 90° direction has the widest attainable range than others, while those along 45° and 0° directions are not changed relatively.

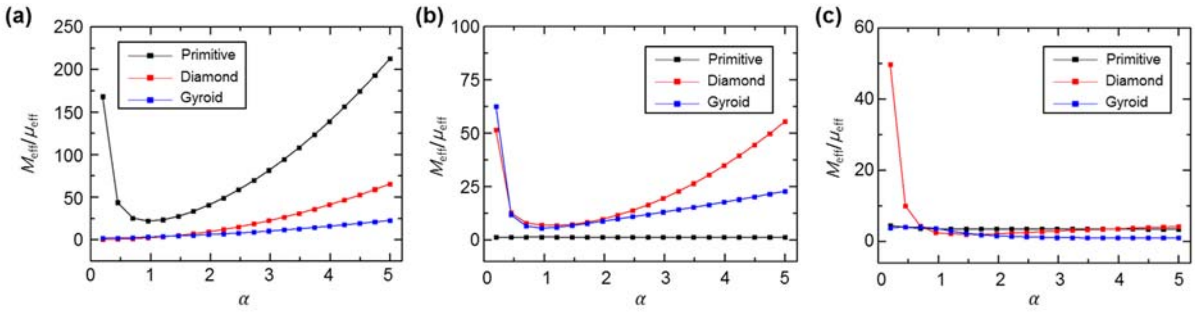

Figure S2. The effect of aspect ratio on the M_{eff}/μ_{eff} ratio along (a) 90° (*z*-direction), (b) 45°, and (c) 0° (*x*-direction).

Boundary conditions in finite element analysis

In order to calculate Young's modulus and shear modulus, we applied the boundary conditions on the surfaces of the structure as shown in figure S3. We applied fixed boundary condition on the bottom of structure. The lateral surfaces of structures were set to be free. Compressive and shear loadings were applied on the top surface of structures along the *z*-direction. The magnitude of applied loading was controlled to be less than 1% that satisfied the small strain approximation.

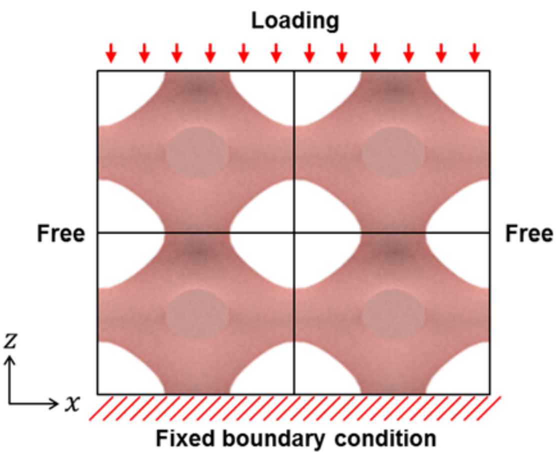

Figure S3. Applied boundary conditions in the finite element analysis.

Reference

1. Stephen Timoshenko, *History of strength of materials: with a brief account of the history of theory of elasticity and theory of structures*. (Courier Corporation, 1953).
